# Medicine dispensing service in primary health care of SUS

**DOI:** 10.11606/S1518-8787.2017051007121

**Published:** 2017-09-22

**Authors:** Silvana Nair Leite, Noemia Liege Maria da Cunha Bernardo, Juliana Álvares, Augusto Afonso Guerra, Ediná Alves Costa, Francisco de Assis Acurcio, Ione Aquemi Guibu, Karen Sarmento Costa, Margô Gomes de Oliveira Karnikowski, Orlando Mario Soeiro, Luciano Soares

**Affiliations:** I Departamento de Ciências Farmacêuticas. Universidade Federal de Santa Catarina. Florianópolis, SC, Brasil; II Universidade do Vale do Itajaí. Itajaí, SC, Brasil; III Departamento de Farmácia Social. Faculdade de Farmácia. Universidade Federal de Minas Gerais. Belo Horizonte, MG, Brasil; IV Instituto de Saúde Coletiva. Universidade Federal da Bahia. Salvador, BA, Brasil; V Faculdade de Ciências Médicas. Santa Casa de São Paulo. São Paulo, SP, Brasil; VI Núcleo de Estudos de Políticas Públicas. Universidade Estadual de Campinas. Campinas, SP, Brasil; VII Programa de Pós-Graduação em Saúde Coletiva. Departamento de Saúde Coletiva. Faculdade de Ciências Médicas. Universidade Estadual de Campinas. Campinas, SP, Brasil; VIII Programa de Pós-Graduação em Epidemiologia. Faculdade de Medicina. Universidade Federal do Rio Grande do Sul. Porto Alegre, RS, Brasil; IX Faculdade de Ciências Farmacêuticas. Pontifícia Universidade Católica de Campinas. Campinas, SP, Brasil; X Faculdade de Ceilândia. Universidade de Brasília. Brasília, DF, Brasil; XI Programa de Pós-Graduação em Saúde e Meio Ambiente. Universidade da Região de Joinville. Joinville, SC, Brasil

**Keywords:** Good Dispensing Practices, Pharmaceutical Services, Primary Health Care, Health Services Research, Brazilian Unified Health System, Boas Práticas de Dispensação, Assistência Farmacêutica, Atenção Primária à Saúde, Pesquisa sobre Serviços de Saúde, Sistema Único de Saúde

## Abstract

**OBJECTIVE:**

To characterize the medicine dispensing services in the primary health care network in Brazil and in its different regions, aiming to promote the access and rational use of medicines.

**METHODS:**

This is a cross-sectional, quantitative study with data obtained from the *Pesquisa Nacional sobre Acesso, Utilização e Uso Racional de Medicamentos* (PNAUM – National Survey on Access, Use and Promotion of Rational Use of Medicines), 2015. Observation visits were carried out in 1,175 dispensing units, and interviews were held with 1,139 professionals responsible for the dispensation of medicines in the dispensing units and 495 municipal coordinators of pharmaceutical services.

**RESULTS:**

More than half (53%) of the units presented a space smaller than 10 m^2^ for dispensing of medicines; 23.8% had bars or barriers between users and dispenser; 41.7% had computerized system; and 23.7% had counters for individual care. Among those responsible for dispensation, 87.4% said they always or repeatedly inform users how to use the medicines, and 18.1% reported developing some type of clinical activity. Isolated pharmacies presented a more developed physical and personal structure than those belonging to health units, but we found no significant differences regarding the information provided and the development of clinical activities.

**CONCLUSIONS:**

There are major differences in the organization models of dispensation between cities, with regional differences regarding the physical structure and professionals involved. The centralization of medicine dispensing in pharmacies separated from the health services is associated with better structural and professional conditions, as in the dispensing units of the South, Southeast, and Midwest regions. However, the development of dispensation as health service does not prevail in any pharmacy or region of the Country yet.

## RESUMO

**OBJETIVO:**

Caracterizar os serviços de dispensação de medicamentos na rede de atenção básica no Brasil e nas diferentes regiões, com vistas ao acesso e a promoção do uso racional de medicamentos.

**MÉTODOS:**

Trata-se de estudo transversal, de abordagem quantitativa, a partir de dados obtidos da Pesquisa Nacional sobre Acesso, Utilização e Uso Racional de Medicamentos, 2015. Foram realizadas visitas de observação em 1.175 unidades de dispensação e entrevistas com 1.139 responsáveis pela dispensação dos medicamentos nas unidades dispensadoras e 495 coordenadores da assistência farmacêutica municipal.

**RESULTADOS:**

Mais da metade (53%) das unidades apresentaram espaço menor que 10 m^2^ para dispensação de medicamentos, 23,8% apresentavam grades ou barreiras entre usuários e dispensador, 41,7% dispunham de sistema informatizado, 23,7% contavam com guichês para atendimento individual. Entre os responsáveis pela dispensação 87,4% afirmaram informar sobre a forma de uso dos medicamentos sempre ou repetidamente, e 18,1% afirmaram desenvolver algum tipo de atividade clínica. As farmácias isoladas apresentavam estrutura física e pessoal mais desenvolvida que aquelas pertencentes a unidades de saúde, mas não houve diferenças significantes quanto às informações prestadas e o desenvolvimento de atividades clínicas.

**CONCLUSÕES:**

Há grandes diferenças de modelos de organização da dispensação entre os municípios, com diferenças regionais quanto à estrutura física e a profissionais envolvidos. A centralização da dispensação de medicamentos em farmácias isoladas dos serviços de saúde está associada a melhores condições estruturais e de profissionais, assim como nas unidades dispensadoras das regiões Sul, Sudeste e Centro-Oeste, no entanto o desenvolvimento da dispensação como serviço de saúde ainda não prevalece em qualquer tipo de farmácia ou região do país.

## INTRODUCTION

The availability of safe, effective, and required medicines, especially those considered essential to face the health problems of developing countries, was the keynote of international recommendations in recent decades, under the general title of “access to essential medicines.” In fact, access to medicines (and their consumption) has increased in all countries, according to a survey of the World Health Organization^11^. In Brazil, the recent results of the population survey on access and use of medicines indicate high levels of access^1^.

Currently, the emphasis has been redirected beyond availability, covering the wider field of the qualification of use of medicines. Such an approach includes strategies so that patients receive the right medicine at the right time, using them properly and with benefit. To this end, health services need to develop activities and employ their capacity and existing resources to promote sustainable solutions that improve the outcomes of patients, which include the organization and qualification of medicine dispensing services as fundamental action^27^.

The term “dispensation” was legally recognized in Brazil, since 1973, as the supply of medicines to consumers in response or not to a medical prescription^5^. Dispensation has nationally and internationally been neglected as object of theoretical reflection, resulting in a simplistic understanding of dispensation of medicines, compliance with legal standards or mere bureaucracy^2^, which is reflected in the practice observed in pharmacies^4^. With the publication of the National Policy of Pharmaceutical Services^14^, the standardization of activities of the pharmacies by the National Sanitary Surveillance Agency (Anvisa^15^), and the recent publication of law no. 13,021^6^, the goal of dispensation starts to approach the needs of society, seeking to transcend the mere supply of medicines to promote their rational use.

In the few studies evaluating dispensing services published in Brazil, the situation described is worrying regarding the quality of services, under the aspects of organization, structure, functionality, and integration with health actions^4^ ^,^ ^19^ ^,^ ^23^.

Research and documents suggest that the complexity of the pharmaceutical practice actions, concerning what the dispensing service can provide – especially regarding the provision of more user-oriented services –, may be limited by working conditions, such as time, infrastructure, and service management^13^ ^,^ ^15^ ^,^ ^27^, causing important and impactful dispensing errors for the patients’ health^12^.

Within the scope of primary health care in the Brazilian Unified Health System (SUS), a series of investments have sought to encourage the development and qualification of pharmaceutical services, with training of teams, resources for structuring the dispensing units, and provision of computerized system^8^ ^,^ ^15^ ^,^ ^22^. Recognizing the current features of dispensing services in the Brazilian primary health care is crucial to evaluate the implementation of the adopted public policies and subsidize future investments.

This article integrates the *Pesquisa Nacional sobre Acesso, Utilização e Promoção do Uso Racional de Medicamentos* (PNAUM – National Survey on Access, Use and Promotion of Rational Use of Medicines) – Services, 2015. It aims to characterize the organization of medicine dispensing services in the primary health care of SUS, to promote access and the rational use of medicines, as well as to identify and discuss the factors that interfere in the qualification of this service in the different regions of the Country.

## METHODS

PNAUM – Services is a cross-sectional, exploratory, evaluative study, consisting of a survey of information in a representative sample of primary health care services in Brazilian cities. Several study populations were considered in the sampling plan, with samples stratified by regions, which constitute the study domains^1^. In this study, we analyzed the in-person interviews conducted with professionals responsible for supplying medicines in primary health care services of SUS, sampled by observation of pharmaceutical services facilities and telephone interviews with those responsible for the municipal Pharmaceutical Services. Data were collected between July and December 2014.

For this analysis, we considered the data stratification by geographic region and by type of dispensing unit. Dispensing units that are isolated, not sharing spaces and structures with other health services, were considered as “isolated pharmacy”; dispensing units located with other health services and belonging to the municipal primary health care network (in basic health units, health centers, joint unit, or other) were considered as “health unit pharmacy.”

Statistical analysis of the data was performed with the SPSS statistical program, extracting the frequencies of the study variables. All analyses considered the sampling weights and structure of the analysis plan for complex samples. For the statistical association analysis, Pearson correlation test was held for categorical variables. The adopted significance level was p < 0.05. Results showed representativeness for the geographic regions of Brazil.

All participants signed the informed consent form. PNAUM was approved by the National Research Ethics Committee of the National Health Council, under opinion no. 398,131/2013. The PNAUM – Services methodology, as well as the sampling process, are described in detail by Álvares et al^1^.

## RESULTS

We analyzed the 1,175 pharmacies/medicine dispensing units of the primary health care network surveyed by PNAUM, as well as the 1,139 interviewed professionals responsible for dispensing medicines in the dispensing units and the 495 municipal pharmaceutical services coordinators.

**Figure 1:**
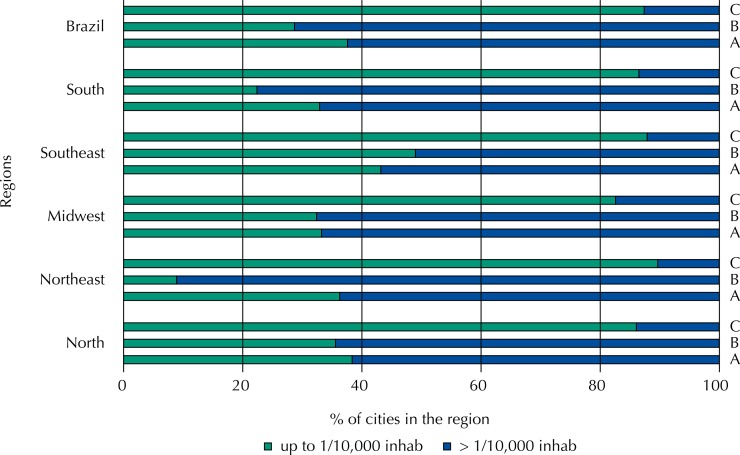
Distribution of medicine dispensing units in Brazilian cities, according to the registered population, in the different regions and in Brazil. National Survey on Access, Use and Promotion of Rational Use of Medicines – Services, 2015.

The distribution of dispensing units by population attended is presented in Figure 1. Most cities maintain more than one pharmacy/dispensing unit by 10,000 inhabitants (62.4%). The frequency of dispensing services in basic health units (UBS) is significantly higher in the Northeast region (91.1%) than in the Southeast (51.0%).

C: specialized centers; B: basic health units; A: isolated pharmacies.

Table 1 presents the characterization of the medicine dispensation of different components and regulatory categories. In Brazil, 52% of cities have dispensed medicines of the Basic Component of Pharmaceutical Services in all their dispensing units (Table 2); this took place in all units of 73.6% of the cities in the Northeast region (significantly higher than in the other regions), in 53.6% in the North, 52.4% in the South, 39.7% in the Midwest, and 35.1% in the Southeast. The medicines of the Specialized Component of Pharmaceutical Services and those dispensed under special control (Ordinance 344/1998) were made available more often in pharmacies from the Southeast region (34.8%; 41.0%, respectively).

**Table 1:** Organization of medicine dispensing in the cities, according to types of medicines and types of dispensing units. National Survey on Access, Use and Promotion of Rational Use of Medicines – Services, 2015.

Type of dispensed medicines	All dispensing units	Some dispensing units	Specialized Centers	Centralized Pharmacy	Pharmaceutical Supply Center	Specialized Pharmacy	Other	Cannot answer
							
	% (95%CI)	% (95%CI)	% (95%CI)	% (95%CI)	% (95%CI)	% (95%CI)	% (95%CI)	% (95%CI)
Basic Component*	52.0 (47.1–56.9)	7.7 (5.3–11.0)	4.5 (2.8–7.4)	28.3 (23.9–33.2)	29.1 (24.6–34.0)	2.0 (1.0–4.2)	6.3 (4.2–9.2)	0.3 (0.1–1.4)
Specialized Component	71.0 (13.8)	29.0 (6.6)	13.0 (2.0)	113.0 (23.3)*	131.0 (28.1)	29.0 (5.1)	78.0 (13.8)	16.0 (3.3)
Ordinance 344	18.4 (14.7–22.7)	6.5 (4.4–9.7)	3.8 (2.2–6.4)	29.8 (25.4–34.7)	31.2 (26.5–36.2)	3.6 (2.1–6.2)	13.3 (10.2–17.1)	0.6 (0.1–2.2)
STD/HIV	5.7 (3.7–8.7)	3.1 (1.8–5.2)	6.2 (4.1–9.2)	9.7 (7.1–13.1)	10.2 (7.5–13.9)	3.30 (2.0–5.5)	19.2 (15.5–23.6)	8.7 (6.2–12.1)
Tuberculosis	15.3 (11.9–19.4)	3.6 (2.2–5.9)	4.5 (2.8–7.4)	14.7 (11.4–18.8)	19.1 (15.3–23.7)	3.1 (1.7–5.4)	24.6 (20.5–29.3)	4.0 (2.4–6.6)

STD: sexually transmitted disease; HIV: human immunodeficiency virus

* p < 0,01

Source: PNAUM – Services, 2015.

**Table 2:** Characterization of the structure and functioning of the medicine dispensing units, according to Brazilian regions. National Survey on Access, Use and Promotion of Rational Use of Medicines – Services, 2015.

Variable	Brazilian regions % (95%CI)					Total

	North	Northeast	Midwest	Southeast	South	
Dispensing area*						
≤ 10 m^2^	60.3 (53.5–66.7)	68.5 (58.5–77.0)	42.7 (30.3–56.1)	45.3 (33.1–58.0)	53.2 (42.1–64.0)	53.8 (48.4–59.1)
> 10 m^2^	39.7 (33.3–46.5)	31.5 (23.0–41.5)	57.3 (43.9–69.7)	54.7 (42.0–66.9)	46.8 (36.0–57.9)	46.2 (40.9–51.6)
Exclusive area for dispensing medicines*						
Yes	60.1 (53.5–66.4)	57.9 (49.6–65.8)	78.1 (64.2–87.6)	72.4 (62.5–80.5)	72.3 (64.1–79.3)	66.2 (61.3–70.7)
Computerized system for the pharmacy						
Yes	17.4 (13.0–22.9)	14.3 (7.9–24.6)	59.0 (44.8–71.9)	60.9 (46.9–73.3)	68.8 (59.8–76.6)	41.7 (36.2–47.5)
Individual service counters with chairs*	23.4 (18.2–29.4)	12.2 (7.1–20.3)	23.6 (15.0–35.2)	38.4 (24.5–54.6)	22.1 (12.6–35.7)	23.7 (17.8–30.8)
Service counter without chairs*	48.6 (42.0–55.2)	38.7 (30.8–47.1)	68.0 (54.4–79.1)	76.7 (67.1–84.1)	73.8 (65.6–80.6)	59.0 (54.0–63.9)
Presence of bars or barriers between dispenser and user*	24.0 (19.2–29.5)	14.3 (8.9–22.1)	41.4 (26.3–58.3)	41.8 (30.3–54.2)	5.4 (3.1–9.0)	23.8 (19.8–28.2)
Hours/week of care*						
Up to 40	79.4 (73.7–84.2)	93 (83.1–97.3)	81.7 (71.7–88.7)	43.2 (31.3–55.9)	63.0 (51.1–73.5)	70.8 (64.5–76.4)
More than 40	20.6 (15.8–26.3)	7.0 (2.7–16.9)	18.3 (11.3–28.3)	56.8 (44.1–68.7)	37.0 (26.5–48.9)	29.2 (23.6–35.5)
Average number of users attended per day*						
Up to 100	91.7 (87.7–94.6)	90.6 (82.8–95.0)	64.7 (51.6–75.9)	47.5 (34.9–60.4)	71.5 (58.7–81.6)	72.6 (65.9)
From 100 to 500	07.7 (04.9–11.7)	1.2 (4.8–17.0)	34.9 (23.7–48.1)	33.2 (22.9–45.5)	22.1 (15.0–31.3)	20.2 (16.2–24.9)
More than 500	0.6 (0.2–1.8)	0.2 (0.1–0.6)	0.4 (0.1–2.7)	19.3 (7.2–42.4)	6.4 (1.0–32.1)	7.2 (2.9–16.8)
Waiting time to be attended < 15 min						
Always or repeatedly	6.4 (3.8–10.8)	4.9 (2.6–9.2)	9.2 (5.1–16.2)	2.9 (1.5–5.7)	8.4 (5.2–13.4)	5.3 (3.9–7.1)
Sometimes or rarely	29.5 (23.8–35.8)	22.2 (14.8–31.8)	27.2 (13.9–46.3)	52.7 (39.6–65.4)	43.5 (32.7–54.9)	36.6 (30.4–43.2)
Never	64.1 (57.4–70.2)	72.9 (63.5–80.6)	63.6 (43.1–80.1)	44.4 (32.4–57.1)	48.1 (37.7–58.6)	58.2 (51.9–64.2)
Exclusive professionals						
Pharmacy*						
Pharmacist	23.2 (18.6–28.5)	22.9 (15.8–31.9)	71.7 (59.7–81.2)	79.0 (70.5–85.5)	40.7 (30.1–52.3)	46.2 (40.9–51.6)
Responsible for the Pharmacy						
Pharmacist*	26.8 (21.9–32.2)	18.5 (11.7–28.1)	66.9 (55.6–76.6)	72.0 (57.3–83.1)	44.8 (34.5–55.6)	43.0 (37.8–48.4)
Information on how to use						
Always or repeatedly	90.8 (86.0–94.1)	93.1 (87.2–96.4)	95.6 (89.7–98.2)	77.2 (55.7–90.1)	90.1 (79.6–95.5)	87.4 (79.3–92.6)
Sometimes or rarely	6.4 (3.8–10.7)	6.0 (2.9–11.9)	4.1 (1.6–10.3)	22.8 (9.9–44.3)	8.1 (3.4–18.3)	11.7 (6.6–20.0)
Never	2.8 (1.2–6.2)	0.9 (0.2–3.8)	0.3 (0.1–1.1)	00 (0.0)	1.7 (0.2–11.4)	0.9 (0.4–2.2)
Conduction of some activity of clinical nature						
Yes	17.9 (13.7–23.1)	12.6 (7.0–21.6)	21.7 (12.1–35.8)	23.0 (15.2–33.0)	19.6 (13.9–26.9)	18.1 (14.5–22.4)

* p < 0,01

Source: PNAUM – Services, 2015.

Regarding structure, most dispensing units observed in the Country presented an exclusive area for dispensation (66.2%). Most units with medicine dispensing service had a space smaller than 10 m^2^ for this practice (53.8%); this area was found in more than 60% of the units in the North and Northeast regions (Table 2), and in 63.2% of health unit pharmacies across the Country (Table 3).

**Table 3:** Characterization of the structure and functioning of the medicine dispensing units, according to the type of dispensing unit. National Survey on Access, Use and Promotion of Rational Use of Medicines – Services, 2015.

Variable	Type of unit	

	Isolated Pharmacy	Center/Joint Unit
	
	% (95%CI)	% (95%CI)
Dispensing area*		
≤ 10 m^2^	26.1 (15.3–40.9)	63.2 (56.3–69.7)
> 10 m^2^	73.9 (59.1–84.7)	36.8 (30.3–43.7)
Exclusive area for dispensing of medicines		
Yes	84.7 (63.2–94.7)	62.0 (56.7–67.0)
Computerized system for the pharmacy*		
Yes	75.5 (61.2–85.7)	35.1 (29.6–41.1)
Individual service counters with chairs*	43.7 (27.7–61.0)	20.5 (14.3–28.6)
Presence of bars or barriers between users and dispenser*	25.8 (13.6–43.5)	23.5 (19.5–28.5)
Hours/week care		
≤ 40	55.2 (37.2–71.9)	73.0 (66.2–78.8)
> 40	44.8 (28.1–62.8)	27.0 (21.2–33.8)
Average number care/day (patients)		
Up to 100	46.9 (31.2–63.3)	78.0 (70.5–84.1)
100–500	40.0 (25.3–56.7)	15.5 (12.3–19.3)
> 500	13.1 (3.5–38.7)	6.5 (2.1–18.7)
Waiting time to be attended >15 min*		
Always or repeatedly	2.1 (0.6–6.6)	6.0 (4.4–8.2)
Sometimes or rarely	62.6 (47.1–75.9)	31.4 (24.9–38.6)
Never	35.3 (22.2–51.0)	62.6 (55.8–69.0)
Exclusive professionals for the pharmacy*		
Pharmacist	85.1 (75.7–91.3)	37.9 (32.1–44.0)
Nurse	5.1 (2.5–9.9)	17.6 (14.4–21.4)
Assistant, technician, other	88.0 (76.0–94.4)	66.3 (61.3–71.0)
Responsible for the pharmacy*		
Pharmacist	87.1 (76.6–93.2)	33.7 (28.5–39.4)
Information on how to use		
Always or repeatedly	85.0 (68.1–93.8)	88.2 (78.3–94.0)
Conduction of some activity of clinical nature		
Yes	26.8 (12.8–47.7)	17.4 (14.2–21.1)

* p < 0,01

Source: PNAUM – Services, 2015.

The presence of bars or barriers between dispenser and users was found in 41.8% of the units in the Southeast and in 41.4% in the Midwest, contrasting with 5.4% of the units in the South. Concerning individual counters for the care, with chairs for staff and users, only 23.7% of the units in the Country had such equipment. Units with counters in which staff and users stand corresponded to the most common pharmacy model in the Country (59.0%) (Table 2).

Most units attend users for up to 40 hours a week, except units in the Southeast and South; in the Southeast, the prevalence of units with more than 40 hours of service a week surpassed 56%. Regarding the average number of users attended per day, the frequency of up to 100 people attended was higher than 90% in the North and Northeast, and lower than 50% in the Southeast (Table 2). The practice of dispensation occurred more frequently in isolated pharmacies, and most of them (located in the South, Southeast, and Midwest) had computerized system for dispensing medicines. The waiting time to be attended was “sometimes” or “rarely” greater than 15 minutes in 62.6% of isolated pharmacies, according to those responsible for the dispensation at the surveyed locations (Table 3).

Dispensing units had record of technical responsibility of pharmacist in 46.2% of the surveyed units in Brazil. The Southeast region presented the highest proportion of technical regularity (79.0%), followed by the Midwest (71.8%) (Table 2), which is a characteristic more common in isolated pharmacies (87.1% of the 116 surveyed pharmacies). These pharmacies also have pharmacists with exclusive dedication to that unit in 85.1% of cases and nurses in 5.1% (Table 3).

In all regions and types of dispensing units in the Country, the conduction of clinical activities was reported in 18.1% of the units. Regarding the provision of information to users on how to use the medicines, 87.4% of those responsible for dispensation stated they always or repeatedly provide information at the time of dispensation. The highest proportion of provision occurring sometimes or rarely (22.8%) was observed in the Southeast, and the highest percentage of professionals who claimed never doing it (2.8%) was observed in the North (Table 2). There was no significant difference between isolated pharmacies and health unit pharmacies regarding the provision of information.

## DISCUSSION

The overcoming of the limited approach of make medicines available in the health services for a more responsible practice regarding the use of therapeutic resources requires understanding dispensation as a health service with premises, structure, workers, and management oriented to the health care of people^15^ ^,^ ^24^. Our results can subsidize the discussions regarding the characteristics of models of organization of services in dispensing units of the cities, punctuating the potential risks to which users are exposed and thus allowing the development of medicine dispensing services and their potential benefits.

Since the management of SUS has the organizational principles of being decentralized and giving the responsibility of primary health care to the cities, the organizational arrangements can be quite varied. The services that dispense medicines in Brazilian cities present great diversity. Little more than half of the cities dispense medicines from the Basic Component of Pharmaceutical Services in all health units, while the rest have other forms of organization such as the concentration of dispensation in centralized pharmacies or reference units. Decentralization associates the distribution of medicines with the provision of other health care offered in the units, while, in centralized cases, accessibility to appointments and other primary health care services is more decentralized than the availability of medicines.

The isolated pharmacy model presents a more developed structural profile, with larger physical spaces, increased office hours, larger numbers of patients attended, higher proportion of units with computerized system, availability of professionals exclusively for this service, and the frequency of technical responsibility of pharmacists. However, the level of information provided to users and the development of clinical activities are low, both in isolated services and in health units. These results suggest that the structural conditions in the pharmacies installed in units specially developed for this purpose are more suitable than those found in pharmacies within other health services, a situation that appears to be a consequence of the establishment of simplistic regulatory standards, as those regulated by Ordinance no. 1,903^16^.

Despite the increased presence of pharmacists and better physical structure, overcoming the model of simple delivery of medicines seems not to have been reached by the centralization of dispensation of medicines. With larger number of users and the existing queues in some of the units observed, the dispensing of the products is still the main service offered. With great demand for the delivery of medicines and isolated from other services and health professionals, pharmacists, in these units, have their possibilities of acting on the health care network constrained^7^.

Even assuming a dispensing operational concept, consisting of routines of prescription validation, separation of medicine, checking of prescription, delivery of medicine, communication with the user for information relevant to the proper use of medicines and care registration, there are strong limitations to the development of medicine dispensation as a health service. The organization of the services, in such a way, can affect the capacity of conducting a professional health care process. The possibility of transcending the pharmaceutical service model focused on local stock management and in the delivery of medicines can be constrained by the material conditions observed^10^ ^,^ ^20^.

Several Brazilian studies report inadequate conditions for the development of dispensing services that aim to promote the rational use of medicines and the care to people^3^ ^,^ ^4^ ^,^ ^26^. Nationally, the work in dispensing units is focused on the accessibility to the product.

Communication with users calls special attention in the presented results. Orientation and education regarding the use and care in using the medicine and promoting the adherence, seeking the best therapeutic results and the risk reduction should be at the heart of the development of a dispensing service^2^. The existence of bars or physical barriers hindering communication was found in more than 40% of the units of the Southeast and Midwest, showing a disturbing scenario. According to Araújo and Freitas^3^, this characteristic can be found in old or new units, possibly as a result of the curative model of health care. The bars in dispensing units also suggest a strong symbolism of the value that permeates the concept of dispensation: custody, protection, and regulation of the medicine product. In this context, one can see how the medicine, as a technology, is more privileged than individuals on certain conceptions of pharmaceutical services^22^. Bars are also a way (sometimes more symbolic than real) of protecting workers from the occasional violence of users dissatisfied with the service received.

The results of such a scenario of dispensing services entail risks to the health of users. Outpatient medicines dispensation could result in errors by up to 22% of cases, some with high risk^9^. Errors are the result of the situation and context in which the dispensation occurs, and they include exchange of products, dosage, or quantities, as well as flaws in the instructions for the correct use of the medicines. Nórden-Hägg et al.^18^ advocate that dispensation in conditions that disperse the professional’s attention (clutter, noise, crosstalk), in work under pressure (excess time or demand for work, queues) and in restricted spaces (small and poorly organized environments, without structure for individual dialogues) lead to the occurrence of dispensing errors and risks to users and professionals.

With the small areas destined for pharmacies in most services, without an area exclusively designed to dispensation and without propitious environment for dialogue and personalized care, the scenario depicted for dispensation in Brazil seems to combine with that described by Nórden-Häag et al.^18^ as conducive to the occurrence of risks for the user. The situation is aggravated by the lack of computerized systems for registration and analysis of the information on users and prescriptions. Situations such as the exemplified by Flynn, Barker, and Carnahan^9^ – of lack of professional guidance about the concomitant use of warfarin and aspirin – are a risk predictor for the user. Such errors are likely to occur frequently in the condition of lack of information about the medicines used by patients, with structural barriers for the communication with them, and in the absence or workload of professionals with clinical reasoning to address the required issues^9^.

In Brazil, the Ministry of Health created a system (Hórus) for information and process management in the pharmaceutical services of the cities, including medicines dispensation. Its aim is to qualify these services and create a national database. The existence of national data allows a broad analysis of the situation and the allowance for decision processes^8^.

The scenario of dispensing units in Brazil evokes the reflection on the need to transform medicine dispensing services in health care points. The situation found is already the result of a period of financial incentives and legal provisions that encouraged the cities to structure pharmaceutical services and primary health care, which allows us to infer that the previous panorama was even worse than the one currently found. For raising dispensation as a model of health service of greater complexity and sociotechnical development^10^ ^,^ ^20^, one must study and define parameters for the structuring of this service and for the technical qualification of the professionals involved.

With this in mind, Soares et al.^24^ propose that dispensation should “consider the access as an attribute; the reception, bond and accountability, the management and pharmaceutical clinic as its components; and the rational use of medicines as a purpose.” The approach requires the integration of clinical and management actions, the multidisciplinary integration of the service with the health system, and the accountability for user access as an attribute inherent to the dispensation of the medicine. Rational use is a goal for being the potential direct result of a properly organized dispensation, with enough complexity to intervene decisively on the course of pharmacotherapy. A sociotechnical development of medicine dispensing units congruent with these principles is also the opportunity to articulate these services with the health promotion practices advocated by SUS, as discussed by Nakamura et al.^17^

Knowledge on the sociotechnical nature of pharmacy in Brazil needs to be object of researches that address SUS as a specific context. The social, technological, and economic dimensions of pharmacy in general, and of the dispensing service in particular, as sociotechnical systems, in the terms of Trist^25^ and Pasmore^21^, need to be reconfigured. One must reorient the work and technology to an integrated organization, in which the pharmacy is a health service that provides pharmaceutical services, and pharmaceutical clinic is a constituent sociotechnical activity of medicine dispensation.

The panorama indicates the need for municipal investments in the creation of suitable conditions so that the increasing access to medicines can be accompanied by their appropriate use. For this, properly organized medicine dispensing services gather opportunities and foundations to decisively affect the health results of medicine users.
